# Clinical Outcomes of the Marginal Ulcer Bleeding after Gastrectomy: As Compared to the Peptic Ulcer Bleeding with Nonoperated Stomach

**DOI:** 10.1155/2012/624327

**Published:** 2012-12-03

**Authors:** Woo Chul Chung, Eun Jung Jeon, Kang-Moon Lee, Chang Nyol Paik, You Suk Oh, Yang Woon Lee, Sang Bae Kim, Kyong-Hwa Jun, Hyung Min Chin

**Affiliations:** ^1^Department of Internal Medicine, The Catholic University of Korea College of Medicine, St. Paul's Hospital, 620-56, Jeonnong 2-dong, Dongdaemun-gu, Seoul 130-709, Republic of Korea; ^2^Department of Surgery, The Catholic University of Korea College of Medicine, Seoul 130-709, Republic of Korea

## Abstract

*Background*. Marginal ulcer is a well-known complication after gastrectomy. Its bleeding can be severe, but the severity has rarely been reported. We aim to evaluate the clinical outcomes of marginal ulcer bleeding (MUB) as compared to peptic ulcer bleeding (PUB) with nonoperated stomach. *Methods*. A consecutive series of patients who had nonvariceal upper gastrointestinal bleeding and admitted to the hospital between 2005 and 2011 were retrospectively analyzed. A total of 530 patients were enrolled in this study, and we compared the clinical characteristics between 70 patients with MUB and 460 patients with PUB. *Results*. Patients with MUB were older (mean age: 62.86 ± 10.59 years versus 53.33 ± 16.68 years, *P* = 0.01). The initial hemoglobin was lower (8.16 ± 3.05 g/dL versus 9.38 ± 2.49 g/dL, *P* = 0.01), and the duration of admission was longer in MUB (7.14 ± 4.10 days versus 5.90 ± 2.97 days, *P* = 0.03). After initial hemostasis, the rebleeding rate during admission was higher (16.2% versus 6.5%, *P* = 0.01) in MUB. However, the mortality rate did not differ statistically between MUB and PUB groups. *Helicobacter pylori*-positive rate with MUB was lower than that of PUB (19.4% versus 54.4%, *P* = 0.01). *Conclusions*. Clinically, MUB after gastrectomy is more severe than PUB with nonoperated stomach. Infection with *H. pylori* might not appear to play an important role in MUB after gastrectomy.

## 1. Introduction

Upper gastrointestinal bleeding remains a common emergency situation. Even though there have been significant improvements in endoscopic and supportive therapies, the overall mortality still remains around 10% and may even reach 35% in hospitalized patients with serious comorbidity [1]. Partial or total gastrectomy has been successfully performed in the treatment of peptic ulcer disease or gastric cancer; however, the potential complications after gastrectomy are numerous. 

One of the major complications is bleeding from marginal ulcer [2]. Marginal ulcer is defined as an ulceration around gastroduodenal or gastrojejunal anastomosis site following partial gastric resection. Its incidence varies from 1% to 16%, and the etiology remains obscure [3, 4]. The possible contributing factors include local ischemia, anastomotic tension, increased gastric acidity, tobacco use, nonsteroidal anti-inflammatory drug use, and chronic irritation caused by the suture materials at the anastomosis [5–8]. However, only a few reports are available on the severity of marginal ulcer bleeding (MUB) after gastrectomy. Furthermore, it is well known that the etiology of peptic ulcer disease is the colonization of *Helicobacter pylori *(*H. pylori*) in the gastric mucosa, whereas the effect of this organism on the remnant stomach following gastrectomy still remains uncertain. 

The aims of this study were to evaluate the differences in the clinical characteristics and the outcomes such as initial hemoglobin, duration of admission, rebleeding rate, and the rate of surgical treatment between MUB and peptic ulcer bleeding (PUB) in patients with nonoperated stomach. Moreover, the association of MUB and *H. pylori *would be elucidated in patients with a history of gastrectomy.

## 2. Methods

### 2.1. Patients

The study was conducted at St. Vincent and St. Paul Hospital, the Catholic University of Korea. The medical records, charts, and the digitalized picture archived images of consecutive patients who were admitted for nonvariceal upper gastrointestinal bleeding between January 2005 and January 2011 were collected. Patients presented with objective evidence of upper gastrointestinal bleeding (hematemesis, melena, or blood from nasogastric aspirates).

All patients underwent an emergency esophagogastroduodenoscopy within 24 hours of initial presentation. No systemic sedative agent was given to any patient. The stigmata of bleeding were classified according to the Forrest classification (Ia, spurting bleeding; Ib, oozing bleeding; IIa, visible vessel; IIb, clot; IIc, black base; III, clear ulcer base) [9]. When the base of ulcer was classified as the Forrest classification I and IIa, the endoscopic treatment was done. In a small number of patients with Forrest IIb, endoscopic treatment was also performed. All patients had a second look endoscopy within 48 hours of initial endoscopic examination. During the second look endoscopy, two biopsy specimens were taken from the antrum (the greater curvature of the mid-antrum) and the corpus (the greater curvature of the midbody) for the histological assessment. The diagnosis of *H. pylori *infection was made by showing histological results—rapid urease test (CLO test, Kimberly-Clark, Utah, United States) or Warthin-Starry silver stain in any of two specimens from the antrum and body. If a case was reported as *H. pylori*-negative, a biopsy for the detection of *H. pylori *was repeated after 4~8 weeks. Alcohol consumption was defined as the consumption of at least 20 g alcohol/day and up to three times/week. Smoking was defined as current smoker.

A total of 530 patients had bleeding ulcers confirmed by endoscopy. Seventy patients had a history of gastrectomy. The outcome of this group of patients was compared to 460 patients without a history of gastrectomy. The patients excluded were all less than 17 years and older than 85 years of age. We excluded procedure-related bleeding (e.g., after gastric polypectomy, endoscopic mucosal resection, or endoscopic submucosal dissection) and patients with a medical comorbidity of serious systemic disease (heart failure, liver cirrhosis, chronic obstructive pulmonary disorder, sepsis, hematologic disorder, etc). However, the patients having diabetes mellitus without complication or hypertension with well-controlled state were included. Individuals with conditions that might have substantial effects on our study results (e.g., serum creatinine > 2.5 mg/dL and total bilirubin > 3.0 mg/dL), and a previous history of peptic ulcer disease, and bleeding associated with malignancy or nonulcer disease (varices, vascular ectasia, Dieulafoy's ulcer, Mallory-Weiss tear, and hemorrhagic erosive gastritis) were excluded. We also excluded the ulcers within first 1 year following gastrectomy.

### 2.2. Methods

The patient age, sex, smoking and alcohol history, initial hemoglobin, duration of admission, endoscopic findings, status of *H. pylori *infection, and clinical outcome were evaluated. Recurrence of bleeding was defined as the objective evidence of bleeding with continuous melena, hematochezia, or the presence of fresh bloody vomitus. When hemodynamic instability (systolic blood pressure <90 mmHg or heart rate >120 beats/minute) had developed or an abrupt drop of more than 2 g/dL of hemoglobin level occurred, these were also defined as a recurrence of bleeding.

### 2.3. Statistical Analysis

The continuous variables were expressed as a mean ± standard deviation and compared using the Student's *t*-test. The categorical variables were expressed as percentages and compared using a Chi-square test with SPSS version 12.0 software (SPSS Korea, Seoul, Korea). A *P-*value of less than 0.05 was regarded as significant.

### 2.4. Ethics Statement

This study was approved by the Institutional Review Board of the Catholic University of Korea (VC12RISI0015).

## 3. Results 

**Figure 1:**
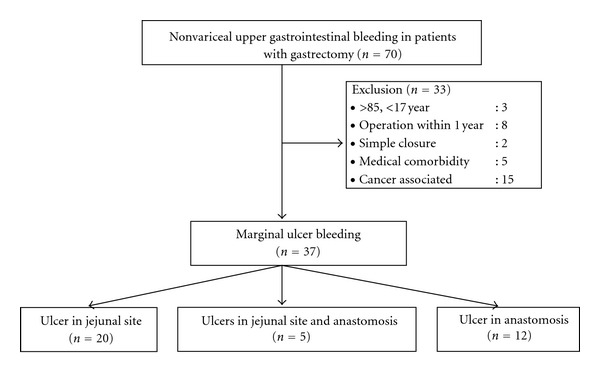
Study enrollment of the marginal ulcer bleeding after gastrectomy.

**Figure 2:**
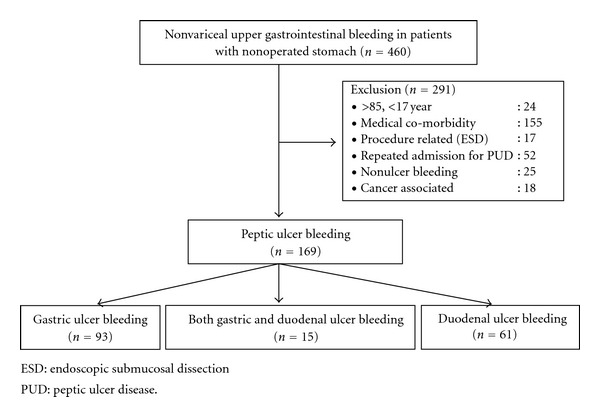
Study enrollment of the peptic ulcer bleeding with nonoperated stomach.

Of the 70 patients with a history of gastrectomy, 33 were excluded due to a history of a gastrectomy within 1 year (*n* = 8), older than 85 years (*n* = 3), simple closure (*n* = 2), significant medical comorbidity (*n* = 5), and bleeding associated with malignancy (*n* = 15) (Figure 1). Of the 460 patients with a nonoperated stomach, 291 were excluded due to extreme age (*n* = 24), a significant medical comorbidity (*n* = 155), procedure-related bleeding (*n* = 17), repeated admissions for peptic ulcer disease (*n* = 52), bleeding from nonulcer disease (*n* = 25), and bleeding associated with malignancy (*n* = 18) (Figure 2). A total of 37 patients with MUB and 169 patients with PUB were enrolled. The characteristics and clinical outcomes of the patients of both groups are shown in Table 1.

**Figure 3:**
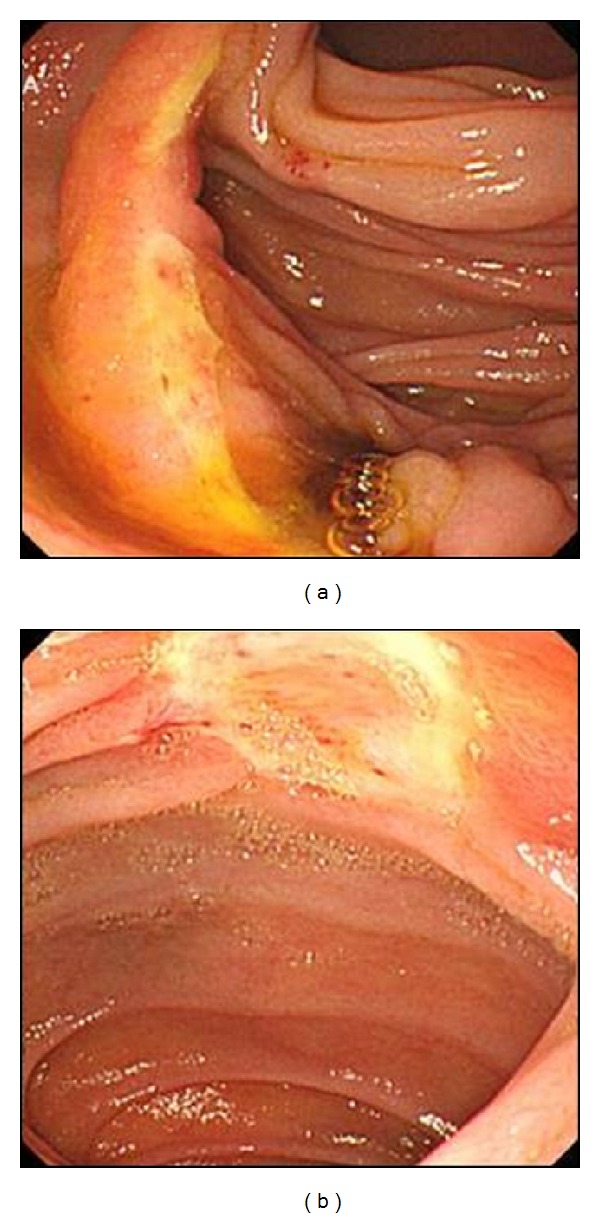
Representative images of the marginal ulcer bleeding in a patient with subtotal gastrectomy. (a) A linear ulceration was observed at anastomosis site. (b) A round ulcer was found on efferent side of Billroth-II anastomosis. And there were red spots on the base of ulcer.

The location of MUB with a history of gastrectomy was jejunal side in 20 patients (54.1%), anastomosis site in 12 (32.4%), and both sites in 5 (13.5%) (Figure 3). In PUB with nonoperated stomach, 93 patients (55.0%) had gastric ulcer bleeding, whereas 61 patients (36.1%) had duodenal ulcer bleeding. Fifteen patients (8.9%) had both gastric ulcer and duodenal ulcer bleeding. 

A total of 24 patients were treated with a gastrectomy for the complications of peptic ulcer disease, whereas 12 patients had an operation for gastric cancer or a gastrointestinal stromal tumor. In the remaining one patient, the cause of gastrectomy was traumatic complication for traffic accident. Five patients of MUB had Billroth-I (B-I) gastroduodenal anastomosis and 32 patients had Billroth-II (B-II) gastrojejunal anastomosis (Table 2).

At the clinical aspect, the patients with MUB were older (mean age: 62.86 ± 10.59 years versus 53.33 ± 16.68 years, *P* = 0.01). The initial hemoglobin at presentation was lower (8.16 ± 3.05 g/dL versus  9.38 ± 2.49 g/dL, *P* = 0.01), and the duration of admission was longer in MUB (7.14 ± 4.10 days versus  5.90 ± 2.97 days, *P* = 0.03). At the initial endoscopic examination, definite bleeding stigmata (Forrest I and IIa) were identified in 15 patients (40.5%) with MUB, whereas 81 patients (47.9%) with PUB (*P* = 0.40). However, the frequency of therapeutic intervention was 37.8% (14/37) in MUB, whereas 56.8% (96/169) in PUB (*P* = 0.03). After an initial hemostasis, the rebleeding rate during admission was higher (16.2% versus 6.5%, *P* = 0.01) in MUB. However, the rate of surgical treatment did not differ statistically between MUB and PUB groups (2.7% versus 4.1%, *P* = 0.66). The infection rates of *H. pylori *in PUB with nonoperated stomach were higher than MUB (54.4% versus 19.4%, *P* = 0.01) after gastrectomy.

According to the cause of gastrectomy, MUB was subclassified into complicated peptic ulcer group and nonulcer group. The MUB with a history of complicated ulcer is 64.9% (24/37). There were no differences of age, initial hemoglobin level, rebleeding rate at admission, and the duration of admission. There was male-predominant feature in MUB with complicated ulcer (*P* = 0.02). The frequency of *H. pylori *infection did not differ significantly between the two groups (Table 2).

## 4. Discussion 

In this study, MUB after a gastrectomy seems to be more severe than PUB with non-operated stomach. This study was unique in three ways. In the first place, we focused on bleeding from benign peptic ulcer (including marginal ulcer), excluding bleeding from malignancy, Mallory-Weiss tear, vascular ectasia, angiodysplasia, and Dieulafoy's ulcer. Secondly, we excluded the patient group of medical comorbidity with systemic disease and extremely old age, because those factors might influence the outcomes of bleeding ulcer. Thirdly, we analyzed the status of *H. pylori *infection with the repeated histological examination.

Previously, it was reported that upper gastrointestinal bleeding would be more severe in surgically treated patients than in nonoperated patients [10]. Although it was a large scaled comparative study, the patients with extremely age and with significant concurrent diseases were not excluded. It would create biased results; therefore, we made concrete criteria to enroll the patients to avoid a bias in this study. Moreover, MUB within 1 year of gastrectomy was excluded from our study. In earlier study, there was a high incidence of marginal ulcer 1 month after surgery (early marginal ulcer), whereas a very low incidence 1 or 2 years after surgery (late marginal ulcer) [11]. It was suggested that different etiological factors were involved in the development of marginal ulcer after gastrectomy. Chronic irritation caused by the suture materials at the anastomosis, use of electrocautery, ischemic injury, and anastomotic stricture may lead to early marginal ulcer formation [4, 8, 11–13]. Although the incidence of late marginal ulcer was low, high output of gastric acid might be a main pathogenesis [12–15]. Therefore, we included only ulcers that developed 1 year or more after surgery, and true marginal ulcer after a gastrectomy that related to the gastric acid could be analyzed.

Since the discovery of *H. pylori*, epidemiologic and clinical studies have provided convincing evidence that *H. pylori *infection is the cause of peptic ulcer disease. It has been accepted that *H. pylori *is a major cause of peptic ulcer [16, 17]. However, in some cases, miscellaneous causes developed peptic ulcer disease without *H. pylori *infection. Until now, it is believed that an imbalance between the protective and aggressive factors acting on the mucosa plays a decisive role in the pathogenesis of a peptic ulcer. Aging and male sex are also considered as risk factors for peptic ulcer disease [18–20]. Our results revealed that patients with MUB were older than PUB with nonoperated stomach. The male-dominant pattern in MUB was less typical than PUB, but there was no statistically significant difference. When MUB was subclassified into complicated ulcer group and nonulcer group, MUB with a history of complicated ulcer was two-thirds of total patients. Except the male-predominant pattern in the ulcer group, there were no differences in the variables for the severity of ulcer bleeding—initial hemoglobin level and duration of admission. In general, the prevalence of peptic ulcers in male patients is markedly higher than that of female patients. This sex difference is still seen in MUB with a history of complicated ulcer. The mechanism by which men have a higher prevalence of peptic ulcer is not clear. Possible mechanisms are suggested as high smoking rate and higher capacity of acid secretion in men. Another possible explanation is that female sex hormones may prevent the development of ulcer. Regretfully, there is no convincing evidence of male preference in ulcer disease. 

Although the reason why MUB after gastrectomy has more severe clinical course remains unclear, it may be related to the iron deficiency anemia, postoperative changes of blood supply, and adhesion with fibrosis of the anastomosis site. The marginal ulcers in Billroth II anastomosis do not have the same mucosa or blood supply system as a peptic ulcer. The marginal ulcer receives blood supply from the jejunal branches of the superior mesenteric artery or gastric branches of the celiac artery [21]. Most of the marginal ulcers are found at the saddle area of the jejunum, and it made angulation among the arteries. The angulation phenomenon supplied the minimum requirement of blood to the mucosal fold of the jejunal saddle area [21]. Moreover, the saddle area of the jejunal loop is the mechanical weak point during food impulsion [10]. The impaired blood flow and the mechanical weak point may partially break the defense mechanism of the jejunal mucosa after injury [2]. Postoperative adhesion with fibrosis just and around the anastomosis site may influence blood supply of a bleeding ulcer site and the ulcer healing mechanism. In our results, more than half of marginal ulcer bleeding were at jejunal side. 

Previously, it has been reported that the presence of a spurting artery is noted more commonly in patients having a history of gastric surgery [10]. However, in our study there was no difference of frequencies of recent bleeding stigmata of ulcer base (Forrest classification I and IIa) between MUB and PUB with nonoperated stomach. It was noteworthy that the frequency of therapeutic intervention in MUB was lower, but the rebleeding rate was higher than PUB with nonoperated stomach. The prediction of rebleeding based on Forrest classification might have a limitation in patients with MUB after gastrectomy, and it needs to be confirmed through further study. A new system for risk stratification of rebleeding would be proposed, and this system might consider both the clinical factors and endoscopic signs in MUB. 

The exact link between *H. pylori *infection and MUB after a gastrectomy is unclear, and *H. pylori *infection is not associated with the severity of MUB. Previously, uncontrolled data have suggested that the frequency of marginal ulcers can be reduced by preoperative screening and treatment of *H. pylori *infection in patients undergoing gastric bypass surgery. This indicates that infection with *H. pylori *may promote marginal ulcer formation [22, 23]. On the contrary, several investigations revealed that the rate of *H. pylori *infection did not play an important role in the pathogenesis of marginal ulcer [24, 25]. In this study, the infection rate of *H. pylori *was significantly low in MUB. However, we could not rule out the possibility that some cases were negative for *H. pylori *at the time of diagnosis of MUB but had suffered from *H. pylori *infection previously. Most of the patients had partial gastrectomy with B-II anastomosis. Bile reflux may result in the interference with colonization by *H. pylori *and spontaneous clearance of the infection. This phenomenon is more typical in B-II than B-I anastomosis [26, 27]. Moreover, biopsy sites suitable for the diagnosis of *H. pylori *infection in the operated stomach have yet to be decided. The potential limitation of our study was the retrospective design; therefore, the possible bias was inherent. Further research with prospective design in this area would be needed. 

In conclusion, MUB in the patients with a history of gastrectomy is more severe than PUB with nonoperated stomach. In contrast with PUB, the association of MUB and the infection rate of *H. pylori *is low.

## Figures and Tables

**Table 1:** Characteristics and clinical outcomes of the patients.

	MUB after gastrectomy (*n* = 37)	PUB with non-operated stomach (*n* = 169)	*P* value
Age	62.86 ± 10.59	53.33 ± 16.68	0.01
Sex (M : F)	23 : 14	130 : 39	0.06
Smoking (yes : no)	18 : 19	81 : 88	0.93
Alcohol (yes : no)	12 : 25	42 : 127	0.15
Initial hemoglobin (g/dL)	8.16 ± 3.05	9.38 ± 2.49	0.01
Duration of admission (day)	7.14 ± 4.10	5.90 ± 2.97	0.03
*H. pylori *infection (%)	9 (19.4)	93 (54.4)	0.01
Ulcer size (cm)	0.84 ± 0.57	1.00 ± 0.71	0.20
Multiplicity of ulcer (%)	16 (43.2)	44 (26.0)	0.04
Ulcer base (Forrest I, IIa) (%)	15 (40.5)	81 (47.9)	0.40
Therapeutic intervention (%)	14 (37.8)	96 (56.8)	0.03
Rebleeding case (%)	6 (16.2)	11 (6.5)	0.01
Surgical treatment (%)	1 (2.7)	7 (4.1)	0.66

Values shown as mean ± SD or No.

**Table 2:** Characteristics and clinical outcomes of MUB according to the history of a peptic ulcer.

	MUB with history of complicated ulcer (*n* = 24)	MUB without history of ulcer (*n* = 13)	*P* value
Age	61.54 ± 12.46	66.69 ± 7.25	0.30
Sex (M : F)	18 : 6	5 : 8	0.02
Smoking (yes : no)	12 : 12	4 : 9	0.25
Alcohol (yes : no)	9 : 15	3 : 10	0.37
Anastomosis			
Billroth-I	3	2	0.81
Billroth-II	21	11	
Initial hemoglobin (g/dL)	8.31 ± 2.10	7.84 ± 3.04	0.68
Duration of admission (day)	6.96 ± 2.70	6.85 ± 2.01	0.93
*H. pylori *Infection (%)	6 (25)	3 (23.1)	0.89
Rebleeding case (%)	3 (12.5)	3 (23.1)	0.40

Values shown as mean ± SD or No.
